# P46 Antibiotic susceptibility response of *adeABC* in the presence of a G-quadruplex DNA molecule

**DOI:** 10.1093/jacamr/dlac004.045

**Published:** 2022-02-16

**Authors:** Chisom Meludu, Christopher O’Kane

**Affiliations:** School of Life Science, Anglia Ruskin University, Cambridge, CB1 1PT, UK; School of Life Science, Anglia Ruskin University, Cambridge, CB1 1PT, UK

## Abstract

**Background:**

G-quadruplexes are secondary nucleotide structures formed from hydrogen binding and stacking of guanine bases at their Watson–Crick and Hoogsteen edges. These structures have confirmed roles in specific genes such as within replication and switching on or off transcription and translation. However, the full potential of these molecular tools has yet to be fully unlocked. The utilization of quadruplexes as molecular tools as well as the further understanding of their mechanistic roles within genetic regulation could lead to the development of novel therapeutic strategies against drug resistance.

**Methods:**

Antibiotic susceptibility profiling studies in 43 clinical *Acinetobacter baumannii* strains revealed MDR properties to ertapenem, meropenem, tetracycline and ciprofloxacin. The role of the efflux pump genes, *adeABC*, to the resistance observed was further confirmed by an inhibitory assay in the presence of PaβN (30 mg/L). Results showed a 97.8% decrease in bacterial growth with ertapenem at a concentration of 125 mg/L when compared with cells without PaβN. However, PaβN is not a viable pharmaceutical compound, as it has toxic properties harmful to life. Therefore, quadruplexes having similar function of inhibiting the mechanism of efflux genes in resistance introduces a substantial alternative in developing a novel antimicrobial therapy. Potential quadruplex forming sequences within the *adeABC* gene of *A. baumannii* (NZ_CP009257.1) using a predictive software tool, QGRS Mapper, were identified. Assembly of the identified sequences was initiated through the instruction of monovalent metal cations (potassium or sodium; 6.8 pH) by heat denaturation assays. Assembly products were further analysed using UV-Vis spectrophotometry, where a thermal difference spectrum (95°C–20°C) was produced, allowing the observation of characteristic quadruplex spectra. A second technique, DNase Assay, was used for additional confirmation of quadruplex assembly, as quadruplexes are resistant to the enzyme's degradation activity.

**Results:**

Five assembled sequences indicated results characteristic of a quadruplex structure formation. A sequence, *ade9*, was then introduced at 1 AU concentration (260 nm) into the bacterial cells of NCTC 12156 strain and incubated with antibiotics at 37°C. It is expected that the introduced assembled quadruplexes would competitively bind to quadruplex-associated-proteins innate within the cells. Where a quadruplex present in cell results in the upregulation of a gene, the introduced quadruples should be observed to result in down-regulation of the efflux pump, increasing susceptibility to the antimicrobial agents. Experiments showed results accepting the proposed hypothesis, with a significant decrease in bacteria growth, indicating a decrease in resistance to the antibiotics.

**Conclusions:**

Resistance of microbes to available therapeutics continues to gain an increased level of concern, posing a threat to life and economy. Identification of the roles of quadruplexes in antimicrobial genes has the potential to unlock a new era for antibiotic therapeutics. Targeting the action of these physiologically active structures may inhibit resistance mechanisms and restore the efficacy of many antimicrobial chemotherapies.

